# Prediction of lower extremity strength by nerve conduction study in cauda equina syndrome

**DOI:** 10.1097/MD.0000000000030124

**Published:** 2022-08-26

**Authors:** Jun-Hyeong Han, Ji-Young Lee, Dong Hyuk Yun, Chang-Won Moon, Kang Hee Cho

**Affiliations:** a Department of Rehabilitation Medicine, School of Medicine, Chungnam National University, Daejeon, Korea; b Institute of Biomedical Engineering, Chungnam National University, Daejeon, Korea.

**Keywords:** cauda equina syndrome, muscle strength, nerve conduction, paralysis

## Abstract

An electrodiagnostic test is more useful than the lower extremity isometric strength test for objectively determining the degree of nerve damage and prognosis in cauda equina syndromes (CES). This study evaluated the correlation between nerve conduction study (NCS) parameters and the lower extremity isometric strength and manual muscle test (MMT) grades.

The isometric strengths of knee extension (KE), ankle dorsiflexion (ADF), and ankle plantarflexion (APF) were measured. NCS parameters, MMT, and isometric strength of femoral, peroneal, and tibial nerves were evaluated, including their correlations with each other. A regression equation between the isometric strength and compound muscle action potential (CMAP) amplitudes was derived and cutoff values were used to confirm boundary values of strength and amplitude between the MMT grades.

KE isometric strength and femoral nerve CMAP amplitude were significantly correlated (*r* = 0.738, *P* < .001). ADF isometric strength and peroneal nerve CMAP amplitude were significantly correlated (tibialis anterior, *r* = 0.707, *P* < .001). KE (*r* = 0.713, *P* < .001), ADF (*r* = 0.744, *P* < .001), and APF (*r* = 0.698, *P* < .001) isometric strengths were correlated with the MMT grades. For the regression curve, the second-order curve was more reasonable than the first-order curve. Cutoff femoral nerve CMAP amplitude and isometric strength cutoff values were ≥2.05 mV and 17.3, respectively, for MMT grades 2 to 3 and 2.78 ± 1.08 and 20.8 ± 9.33, respectively, for grade 3.

The isometric strengths of the KE, ADF, and APF and the CMAP amplitude of the electrophysiologic parameters were correlated in CES patients and a significant correlation with MMT grade was also identified. Accordingly, it is possible to identify the precise neurological condition, objectively evaluate the degree of paralysis and disability, and determine the quantitative muscle strength from MMT in order to establish an appropriate rehabilitation treatment plan.

**What Was Known/What This Paper Adds**

**What Was Known**

An electrodiagnostic test is more beneficial than the lower extremity isometric strength test for objectively determining the degree of nerve damage in cauda equina syndrome patients.

**Key points**

The isometric strengths of the knee extensor, ankle dorsiflexor, and ankle plantarflexor muscles and the amplitude of the compound muscle action potential were correlated in patients with cauda equina syndrome. A significant correlation with the manual muscle test grade was also identified. Based on these findings, it is possible to objectively evaluate the degree of paralysis and disability as well as determine quantitative muscle strength for establishing a rehabilitation plan.

## 1. Introduction

Cauda equina syndrome (CES) results from damage to a neuromuscular bundle below the spinal cone in the spinal canal. The neuromuscular bundle includes motor nerve fibers, which control skeletal muscle, sensory nerve fibers of the skin, and parasympathetic nerves in the sacral region.^[1]^ The 3 common signs of CES include saddle anesthesia, urinary retention, and lower extremity weakness. As peripheral nerve function is lost, the bulbospongiosa reflex, perianal reflex, and tendon reflex of the lower extremities are also lost.^[2]^ The most common cause of CES is compression due to a herniated disc, followed by traumatic fracture, tumor, infection, stenosis, subdural hematoma, inflammation, and vascular causes.^[3]^ The incidence rate of CES is 1 in 33,000 to 100,000 people, and it is common among men in their 30s and 40s. CES has been reported to occur in 4 of 10,000 patients with low back pain.^[4]^ When CES is suspected, magnetic resonance imaging (MRI) should be immediately performed to identify structural lesions. Since spinal canal compression is the primary cause, urgent surgical decompression is needed in acute CES as it may cause neurological paralysis if left untreated.^[5]^

Unlike other spinal cord syndromes, CES causes peripheral nerve damage due to neuromuscular injury and has a high possibility of recovery through nerve regeneration.^[6]^ To determine the prognosis of the peripheral syndrome, it is important to understand the degree of damage to the peripheral nervous system. Prognostic factors for CES include the lower extremity isometric strength score and the American Spinal Injury Association Impairment Scale. However, these methods of evaluation are not considered absolute or objective since they require active patient cooperation. Therefore, alternative objective tests such as nerve conduction studies (NCSs) and electromyography (EMG) are widely used to diagnose and evaluate the severity of nerve damage.^[7]^ This is because a decrease in the compound motor action potential (CMAP), an NCS parameter, indicates the loss of motor neuron axons, muscle weakness, and the severity of the damage.^[8]^ Hollie and Power reported that the decreased CMAP amplitude measured in the first dorsal interosseous muscle was superior to the decrease in the nerve conduction velocity inching test performed at the elbow joint for predicting preoperative grip strength and key pinch test in cubital tunnel syndrome patients. Furthermore, CMAP amplitude is an important indicator of the severity of cubital tunnel syndrome, and CMAP can be used to decide surgical interventions.^[9]^ Sasaki et al reported that the CMAP for abductor pollicis brevis and second-finger sensory nerve action potentials (SNAPs) measured by palmar stimulation before surgery in carpal tunnel syndrome could predict postsurgery improvement. Another study stated that the CMAP amplitude was an indicator of the number of remaining axons, in which functional recovery could be predicted, and that Bland scale improvement could be expected if most axons remain.^[10]^ Although there have been several studies on single-nerve damage, there are few studies on the prognosis and relationship between NCS, CMAP, and muscle strength in multiple root neuropathy with damaged nerve root bundles, such as in CES. This study aims to present a guideline that can be used for the objective evaluation of the degree of lower extremity paralysis caused by CES or the degree of lumbar nerve root injury via NCS by analyzing the correlation between the muscle strength of knee extensor (KE), ankle dorsiflexor (ADF), and ankle plantarflexor and NCS variables in patients with CES.

## 2. Methods

This cross-sectional study included 96 patients aged ≥18 years who were admitted to our institution from April 2019 to April 2021 and had a CES history for ≥3 weeks. The study excluded cases with damage to the spinal cone or more, such as spinal cord injury or brain injury; peripheral neuropathy caused by diabetes or chemotherapy; industrial accidents or other secondary gains; and local peripheral nerve damage to the lower extremity due to fracture or entrapment neuropathy. In addition, patients with impaired communication, such as cognitive decline, were also excluded. This study involved a retrospective review of medical records and was approved by the Institutional Review Board of Chungnam National University Hospital, Republic of Korea (IRB No. 2018-03-057). The patients have given written informed consent.

Manual muscle testing (MMT) was performed by the same experienced physiatrist and evaluated in 6 grades (0–5). Isometric contractile strength (N-m) was measured for KE, dorsiflexion, and plantar muscle strength (System 4 Pro™, Biodex Medical Systems). KE was measured at 30° flexion, and dorsiflexion and plantarflexion were measured in the neutral position. The maximum muscle strength was measured 3 times. To ensure accuracy, results measured within 15% of the maximum muscle strength were used for the remaining 2 measurements, in addition to the maximum strength. Dantec Keypoint G4 (Natus, Middleton, WI), with the Neuroline 715 electrode (Ambu, Ballerup, Denmark) was used for NCS. The electrode was 32 × 22 mm in size and was made of Ag/AgCl; a solid gel was used as an adhesive material.

The peroneal nerve (to extensor digitorum brevis [EDB]) and the tibial nerve (to abductor hallucis [AH]) were used to analyze motor nerve conduction, whereas the sural nerve was used to analyze sensory nerve conduction. When necessary, in order to maintain skin temperature close to room temperature, the lower limb was warmed using a heat pack. CMAP and SNAP onset latencies were measured at the initial point of the negative phase of the potential. The CMAP amplitude was measured from baseline to the negative peak, and the SNAP amplitude was measured from the first negative peak to the following positive peak. The settings for the recordings of the motor studies were as follows: sensitivity, 3–5 mV/division; low-frequency filter, 10 Hz; high-frequency filter, 10 kHz; and sweep speed, 5 ms/division. The settings for the recordings of the sensory study were as follows: sensitivity, 20 mV/division; low-frequency filter, 10 Hz; high-frequency filter, 2 kHz; and sweep speed, 2 ms/division.

### 2.1. Nerve conduction studies

#### 2.1.1. Femoral nerves.

The subjects were positioned supine, and the active electrode was placed on the vastus medialis muscle belly, 4 cm above the patella margin. The reference electrode was placed on the patella, and the ground electrode was placed on the dorsal side of the foot. Nerve stimulation was applied to the inguinal ligament, lateral to the femoral artery.

#### 2.1.2. Peroneal nerves.

The subjects were positioned supine, and the active electrode was placed on EDB. The reference electrode was placed on the fifth metatarsophalangeal joint, and the ground electrode was placed on the dorsal side of the foot. Distal nerve stimulation was applied 8 cm proximal to the active electrode, just lateral to the tibialis anterior (TA) tendon. Proximal nerve stimulation was applied just below the fibular head. Afterwards, the active electrode was placed on the TA muscle belly, and the reference electrode was placed on the medial malleolus. Stimulation was applied just below the fibular head.

#### 2.1.3. Tibial nerves.

Subjects were positioned supine, and the active electrode was placed on AH, 1 cm behind and 1 cm below the navicular tubercle. The reference electrode was placed on the large toe. Distal nerve stimulation was applied with the cathode 8 cm proximal to the active electrode, posterior to the medial malleolus. Proximal nerve stimulation was applied to the crease of the popliteal fossa between the lateral third and the medial two-thirds. Next, the active electrode was placed on the gastrocnemius medialis (GCM) muscle belly, and the reference electrode was placed on the achilles tendon. Stimulation was applied to the popliteal fossa crease between the lateral third and the medial two-thirds.

#### 2.1.4. Sural nerves.

The active electrode was placed below and posterior to the lateral malleolus, and the reference electrode was placed 4 cm distally. Stimulation was applied approximately 14 cm proximal to the active electrode, slightly lateral to the midline, in the lower third of the calf.

#### 2.1.5. Superficial peroneal nerves.

The active electrode was placed 1–2 cm medial to the lateral malleolus, and the reference electrode was placed 4 cm distally. Stimulation was applied approximately 14 cm proximal to the active electrode, in the lower third of the lateral calf.

#### 2.1.6. F-wave.

The subjects were positioned supine, and the active electrode was placed on AH, 1 cm behind and 1 cm below the navicular tubercle. The reference electrode was placed on the large toe. Distal nerve stimulation was applied with the cathode 8 cm proximal to the active electrode, posterior to the medial malleolus. The anode was distal. Ten supramaximal stimuli were applied.

#### 2.1.7. H-reflex.

The subjects were positioned supine, and the active electrode was placed on the GCM muscle belly, and the reference electrode was placed on the Achilles tendon. Stimulation was applied to the popliteal fossa crease between the lateral third and the medial two-thirds. The anode was distal. The stimulation intensity was selected to produce maximal H-reflex amplitude together with minimal M-wave as seen in the earlier phase

#### 2.2. Statistics.

Pearson correlation analysis was performed to determine the correlation between strength and all NCS variables. A regression analysis of CMAP amplitude and strength among the NCS variables was performed. Cutoff values were obtained by drawing receiver operating characteristic (ROC) curves to divide sections according to the MMT grade of amplitude and strength. The cutoff value was defined as the value with the highest sum of sensitivity and specificity in the ROC curve. The mean ± SD values of CMAP amplitude and strength according to the MMT grade for each nerve were compared. SPSS 26.0 (IBM, Armonk, NY) was used for the statistical analysis. Statistical significance was set at *P* < .001.

## 3. Results

**Figure 1.**
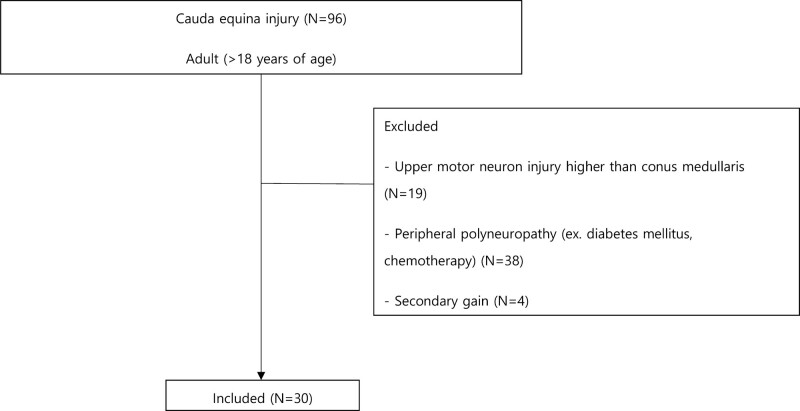
Study flowchart of the subjects with cauda equine syndrome.

Ninety-six patients with CES were selected, among whom, 19 had spinal cord injury, 38 patients had peripheral neuropathy, 5 patients had lower extremity fractures, and 4 patients with secondary gain were excluded. Ultimately, 30 patients were included in the analysis (Fig. 1). The mean age of the subjects (18 men, 12 women) was 62.07 ± 18.38 years (Table 1).

**Table 1:** Demographic findings of the included patients.

	N: 30
Sex (n)	M:18 F:12
Age (yr)	62.07 ± 18.38
MMT knee extensor	N:18 G:22 F:7 P:5 T:4 Z:4
MMT ankle dorsiflexor	N:8 G:16 F:14 P:9 T:9 Z:4
MMT ankle plantarflexor	N:11 G:22 F:12 P:5 T:6 Z:4
IST knee extensor	36.27 ± 28.26
IST ankle dorsiflexor	11.85 ± 9.89
IST ankle plantarflexor	35.42 ± 30.7
Amplitude of femoral nerve (mV)	4.24 ± 3.10
Amplitude of peroneal nerve (TA) (mV)	2.46 ± 1.89
Amplitude of tibial nerve (GCM) (mV)	4.22 ± 3.17

F = fair (3) grade, G = good (4) grade, GCM = gastrocnemius medialis, IST = isometric strength test, MMT = manual muscle test, N = normal (5) grade, P = poor (2) grade, T = trace (1) grade, TA = tibialis anterior, Unit = N-m, Z = zero (0) grade.

Table 2 shows the correlations between electrophysiological variables and isometric muscle strength. KE isometric strength was significantly correlated with the femoral nerve CMAP amplitude (*r* = 0.738, *P* < .001). ADF isometric strength and peroneal nerve CMAP amplitude were significantly correlated (TA, *r* = 0.707, *P* < .001; EDB, *r* = 0.580, *P* < .001). Ankle plantarflexor isometric strength was significantly correlated with the tibial nerve amplitude (GCM, *r* = 0.687, *P* < .001; AH, *r* = 0.511, *P* < .001). The AH CMAP amplitude showed moderate correlation, but the GCM CMAP amplitude showed a higher correlation coefficient. No other significant correlation was observed between the latency and velocity of motor nerves, each variable of the sensory nerves, and the isometric strength of each muscle. There was no significant correlation between F-wave latency or H-reflex latency and isometric strength. KE (*r* = 0.713, *P* < .001), ADF (*r* = 0.744, *P* < .001), and ankle plantarflexor (*r* = 0.698, *P* < .001) isometric strength were each significantly correlated with MMT grade (Table 3). To reflect the correction for weight, the Pearson correlation coefficient was compared by dividing the patient’s muscle strength by both weight and body mass index (BMI; Table 4). The comparison showed a very slight increase in the correlation coefficient between weight and BMI-corrected values and amplitude compared to noncorrected values for weight and BMI.

**Table 2:** Correlation coefficient between parameters of nerve conduction study and isometric strength.

IST	Am FN	Amp PN (TA)	Amp PN (EDB)	Amp TN (GM)	Amp TN (AH)	FN Lat	PN Lat	EDB Lat	TN Lat
KE	0.738**	0.413	0.381	0.237	0.302	0.021	−0.011	0.031	−0.166
ADF	0.376	0.707**	0.580**	0.406	0.125	0.149	0.116	−0.267	−0.189
APF	0.410	0.337	0.240	0.687**	0.511**	0.092	0.044	−0.274	−0.234
IST	AH Lat	CV (PN)	CV (TN)	SPN Lat	SN Lat	SPN Amp	SN Amp	Fw lat	Hr Lat
KE	−0.092	−0.068	−0.086	−0.215	−0.182	0.225	0.200	−0.204	−0.133
ADF	−0.177	−0.212	−0.248	−0.106	−0.158	0.155	0.237	−0.017	0.209
APF	−0.042	−0.161	−0.130	0.093	−0.068	−0.134	−0.114	0.190	−0.171

A = ankle, ADF = ankle dorsiflexor, AH = abductor hallucis, Amp = amplitude, APF = ankle plantarflexor, CV = conduction velocity, EDB = extensor digit brevis, FH = fibular head, FN = femoral nerve, Fw = F-wave, GM = gastrocnemius medialis, Hr = H-reflex, IST = isometric strength test, KE = knee extensor, Lat = latency, PF, popliteal fossa, PN = peroneal nerve, SN = sural nerve, SPN = superficial peroneal nerve, TA = tibialis anterior, TN = tibial nerve.

** *P* value < .001.

**Table 3:** Correlation coefficient between manual muscle test and isometric strength.

IST	MMT (KE)	MMT (ADF)	MMT (APF)
KE	0.713**	0.368	0.392
ADF	0.282	0.744**	0.408
APF	0.377	0.389	0.698**

ADF = ankle dorsiflexor, APF = ankle plantarflexor, IST = isometric strength test, KE = knee extensor, MMT = manual muscle test.

** *P* value < .001.

**Table 4:** Correlation coefficient between electrophysiologic parameters and isometric strength.

IST	CMAP amp vs strength	CMAP amp vs strength/kg	CMAP amp vs strength/BMI
KE	0.738**	0.756**	0.766**
ADF	0.707**	0.735**	0.737**
APF	0.687**	0.704**	0.694**

ADF = ankle dorsiflexor, APF = ankle plantarflexor, BMI = body mass index, CMAP = compound motor action potential, IST = isometric strength test, KE = knee extensor.

** *P* value < .001.

**Figure 2.**
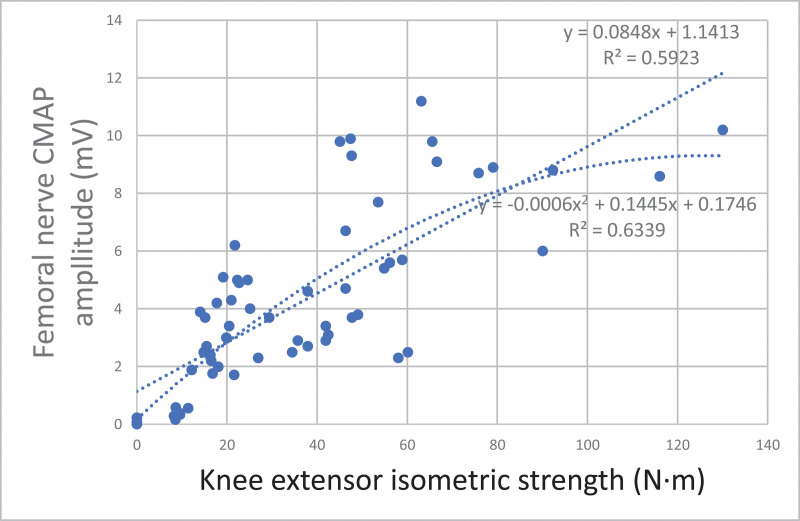
Regression equation between isometric strength and femoral nerve CMAP amplitudes. CMAP = compound muscle action potential.

**Figure 3.**
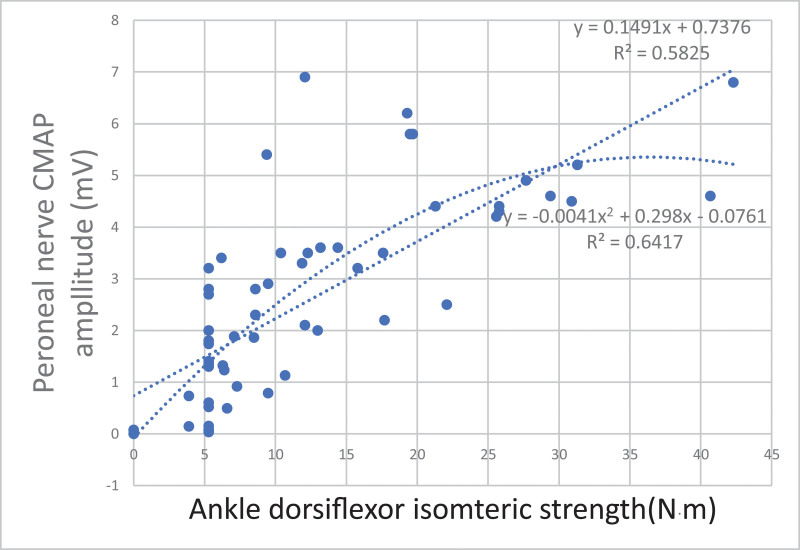
Regression equation between isometric strength and peroneal nerve CMAP amplitudes of tibialis anterior. CMAP = compound muscle action potential.

**Figure 4.**
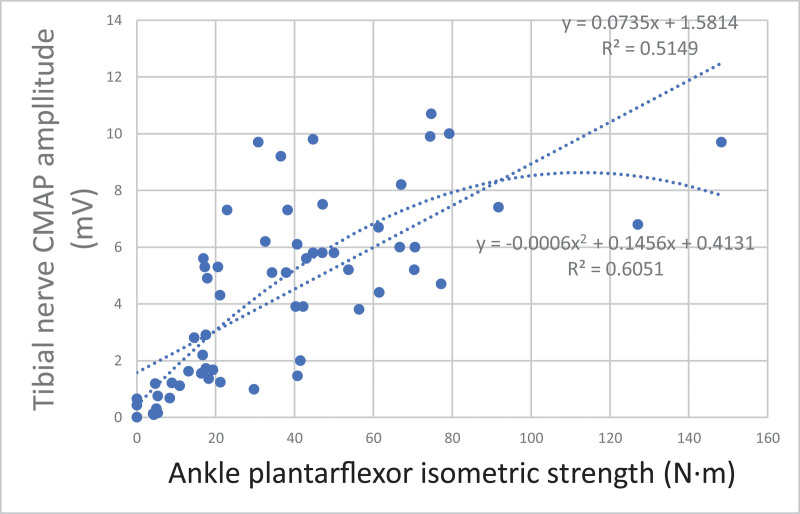
Regression equation between isometric strength and tibial nerve CMAP amplitudes of gastrocnemius medialis. CMAP = compound muscle action potential.

Among the lower extremity NCS variables, regression analysis was performed for motor nerve CMAP amplitude, which was significantly correlated with isometric strength, to estimate the relationship between the variables (Figs. 2–4). The first-order curve estimation equation for femoral nerve CMAP amplitude and KE isometric strength was *Y*(Amp) = 0.0848*X(strength) + 1.1413, and the *R*^2^ value was 0.592. The second-order curve estimating equation was *Y* = −0.0006*X2 + 0.1445*X + 0.1746, and the *R*^2^ value was 0.633 (Fig. 2). The first-order curve estimating equation for the peroneal nerve CMAP amplitude and the ADF isometric strength was *Y* = 0.1491*X + 0.7376, and the *R*^2^ value was 0.582. The second-order curve estimating equation was *Y* = −0.0041*X2 + 0.298*X−0.0761, and the *R*^2^ value was 0.641 (Fig. 3). The first-order curve estimation equation for the tibial nerve CMAP amplitude and ankle plantarflexor isometric strength was *Y* = 0.0735*X + 1.5814, and the *R*^2^ value was 0.515. The second-order curve estimating equation was *Y* = −0.0006*X2 + 0.1456*X + 0.4131, and the *R*^2^ value was 0.605 (Fig. 4).

Cutoff values were obtained by drawing ROC curves to divide sections according to the MMT grade of amplitude and strength. The cutoff value was defined as the value with the highest sum of sensitivity and specificity in the ROC curve (Table 5). For example, for grade 3 in MMT, the cutoff value for CMAP amplitude was predicted to be ≥2.05 mV for the femoral nerve, ≥1.73 mV for the peroneal nerve, and ≥2.30 mV for the tibial nerve. When a patient with CES was grade 3 in MMT, isometric strength was predicted as 17.3, 7.65, and 17.7 N-m, respectively, for the nerves.

**Table 5:** Cutoff value for CMAP amplitude, IST, and IST per kg with respect to MMT grades.

MMT CMAP (mV)	Z-T	T-P	P-F	F-G	G-N
Femoral. n	0.21	1.04	2.05	3.15	5.05
Peroneal n. (TA)	0.18	0.86	1.73	2.40	3.80
Tibial n. (GCM)	0.45	1.02	2.30	3.90	5.50
MMT IST (N·m)	Z-T	T-P	P-F	F-G	G-N
Knee extensor	6.45	11.8	17.3	26.65	43.8
Ankle dorsi	1.95	5.75	7.65	13.65	22.7
Ankle plantar	4.60	8.65	17.7	31.15	49.95
MMT IST(N·m)/kg	Z-T	T-P	P-F	F-G	G-N
Knee extensor	0.10	0.18	0.29	0.43	0.73
Ankle dorsi	0.03	0.08	0.12	0.26	0.36
Ankle plantar	0.07	0.14	0.28	0.50	0.83

CMAP = compound motor action potential, F = fair, G = good, GCM = gastrocnemius medialis, IST = isometric strength test, MMT = manual muscle test, N = normal, P = poor, T = trace, TA = tibialis anterior.

The mean ± SD values of CMAP amplitude and strength according to the MMT grade for each nerve were compared (Table 6). They were calculated from trace to normal grades, and through MMT, the predictive values for an electrodiagnostic test and strength were determined. For grade 3 (fair), the mean ± SD of the femoral, peroneal, and tibial nerves was 2.78 ± 1.08, 1.98 ± 1.03, and 3.26 ± 2.18 mV, respectively. Isometric strength was predicted as 20.8 ± 9.33, 9.28 ± 5.28, and 23.85 ± 10.69 N-m, respectively, for the above nerves.

**Table 6:** CMAP amplitude, IST (N·m), and IST (N·m) per kg according to MMT grades.

MMT CMAP (mV)	T	P	F	G	N
Femoral. n	0.45 ± 0.20	1.88 ± 1.06	2.78 ± 1.08	4.06 ± 1.72	6.94 ± 2.81
Peroneal n. (TA)	0.42 ± 0.26	1.22 ± 0.81	1.98 ± 1.03	3.25 ± 1.47	5.03 ± 1.98
Tibial n. (GCM)	0.72 ± 0.32	1.68 ± 1.01	3.26 ± 2.18	4.65 ± 2.56	7.10 ± 2.06
MMT IST (N·m)	T	P	F	G	**N**
Knee extensor	9.86 ± 1.41	15.4 ± 3.34	20.8 ± 9.33	32.79 ± 17.22	67.03 ± 24.21
Ankle dorsi	5.12 ± 0.49	6.83 ± 1.13	9.28 ± 5.28	18.7 ± 8.67	27.36 ± 8.40
Ankle plantar	6.33 ± 1.78	12.05 ± 6.13	23.85 ± 10.69	41.33 ± 18.17	76.15 ± 23.39
MMT IST (N·m)/kg	T	P	F	G	**N**
Knee extensor	0.14 ± 0.02	0.24 ± 0.06	0.34 ± 0.15	0.54 ± 0.25	0.96 ± 0.37
Ankle dorsi	0.07 ± 0.03	0.10 ± 0.02	0.14 ± 0.06	0.29 ± 0.12	0.40 ± 0.10
Ankle plantar	0.11 ± 0.03	0.21 ± 0.14	0.38 ± 0.16	0.67 ± 0.29	1.09 ± 0.41

CMAP = compound motor action potential, F = fair, G = good, GCM = gastrocnemius medialis, IST = isometric strength test, MMT = manual muscle test, N = normal, P = poor, T = trace, TA = tibialis anterior.

## 4. Discussion

This prospective study revealed a significant relationship between the CMAP amplitude of NCS and isometric strength and MMT grades for the KE, ADFs, and plantarflexors. Since the cauda equina, the peripheral nerve root bundle in the spinal canal, has less connective tissue, with the nerve fibers lacking room for distension, it is vulnerable to physical distortion such as compression or infection.^[11]^ Peripheral nerves undergo Wallerian degeneration, where axonal degeneration and demyelination occur 3 to 5 days after injury.^[12]^ With mild damage, only focal demyelination occurs at the injured site and a conduction block occurs, but with severe damage, the axon is degraded by the action of Schwann cells and macrophages in the distal part of the damage.^[13]^ Axon degradation results in a reduction in the motor unit of the corresponding muscle, and a decrease in the motor unit indicates a decrease in CMAP amplitude.^[14]^ Thus, in electrodiagnostic tests, the amplitude reflects the total number of nerve axons and the density of dominant depolarized muscle fibers.^[15]^ Moreover, as the motor unit number can be predicted by dividing the CMAP amplitude by single-fiber EMG, CMAP amplitude has a significant correlation with the number of axons.^[16]^

Muscle strength is a function of the recruitment, frequency, and synchronization of the motor unit, which is the basic unit of exercise. Motor unit recruitment refers to the number of motor units and muscle types, frequency refers to the firing rate, and synchronization refers to the temporal coincidence of 2 or more motor units. Action potentials (APs) of 1 motor unit are called MUAPs. Since CMAP amplitude is the summation of surface-recorded MUAPs, it can be inferred that CMAP amplitude is correlated with strength.^[17]^ Lippold et al first reported that the isometric strength of the ankle plantarflexor had a linear relationship with the integrated EMG signal measured in GCM. Since then, several studies have investigated the relationship between peripheral nerve electrophysiological parameters and muscle strength.^[18–20]^ Regarding other mononeuropathy studies, a study by Seo et al reported that the amplitude and area of CMAP in patients with common peroneal and tibial nerve injuries was strongly correlated with maximal muscle strength in dorsiflexion and plantarflexion, and that the ratio of the CMAP amplitudes (P grade of 0.2, F grade of 0.43, and G grade of 0.57 compared to the unaffected side) was expressed differentially according to the MMT.^[21]^ Won et al reported that the amplitude of CMAP, compared to the uninjured side of patients with common peroneal nerve injury, showed a correlation with dorsiflexion muscle strength and the ratio of MMT and amplitude (T grade of 0.13, P grade of 0.35, F grade of 0.61, and G grade of 0.77 compared to the unaffected side).^[22]^ In the current study, a significant change in the CMAP amplitude of each motor nerve was observed according to the degree of injury, as demonstrated by the degree of muscle strength reduction. Among them, TA and GCM CMAP amplitudes of the peroneal and tibial nerves, respectively, showed a stronger correlation with isometric strength than the EDB and AH CMAP amplitudes. The CMAP amplitude of TA showed a high positive correlation, and the CMAP amplitude of EDB showed a relatively low, moderate correlation (0.9–1: very high; 0.7–0.9: high; 0.5–0.7: moderate; 0.3–0.5: low; 0–0.3: negligible).^[23]^ The AH CMAP amplitude showed moderate correlation, but the GCM CMAP amplitude showed a higher correlation coefficient. This is because the muscles involved in ankle dorsiflexion and plantarflexion are TA and GCM, which are located more proximally than EDB and AH. Nevertheless, in this study, unlike previous studies, it was not possible to evaluate nerve damage, such as CES damage, in both lower extremities compared to the unaffected side.

During NCS, as sensory nerves are relatively small in size, the APs of all axons of the nerve can be recorded. However, for motor nerves, it is impossible for all muscle APs to be recorded because many muscle fibers are far from the recording electrode. While the APs of most muscle fibers can be recorded for small muscles, only a fraction of the total number of muscle fibers can be recorded for large muscles, as most muscle fibers are located far away. Therefore, the amplitude is proportional to the density of the axon or muscle fiber. Even when very few axons remain, as in the case of previous poliomyelitis, reinnervation occurs, and CMAP can recover to the normal range.^[15]^ People have different muscle sizes and thicknesses, to convert (correct) muscle mass into density for comparing muscle strength between men, with more muscles, and women, with relatively fewer muscles. In this study, the Pearson correlation coefficient was compared by dividing muscle strength by both weight and BMI (Table 4). The comparison showed very slight difference in the correlation coefficient between weight and BMI adjusted for muscle strength and amplitude. For an accurate comparison of muscle mass, it is necessary to conduct research that reflects individual whole-body muscle mass measured by dual-energy X-ray absorptiometry.

In this study, a regression curve was plotted to estimate the isometric strength according to the CMAP amplitude for each nerve. Electrophysiological tests are most crucial for evaluating peripheral nerve function. In radiculopathy, MRI can identify nerve compression, but electrophysiological tests are required to determine the degree of nerve damage caused by compression.^[7]^ Moreover, it is important to objectively assess the degree of nerve damage when evaluating industrial accidents or detecting disability and to identify whether a secondary gain or conversion disorder exists. The correlation between electrophysiological tests and muscle strength can be used to determine the degree of nerve damage objectively. Seo et al^[21]^ reported that the amplitude and area of CMAP in patients with common peroneal and tibial nerve injuries showed a linear correlation with maximal isometric muscle strength in dorsiflexion (*r* = 0.690, *P* < .001) and plantarflexion (*r* = 0.670, *P* < .001). Won et al^[22]^ suggested that the amplitude of CMAP showed a linear correlation with dorsiflexion isometric strength only in the peroneal nerve (*r* = 0.790, *P* < .001). In this study, correlation coefficients were higher than the values reported by Seo et al in both the peroneal and tibial nerves but, were lower than the values by Won et al in the peroneal nerve. Additionally, a regression equation was not reported in any of these studies. This study determined the first-order linear relationship and an additional second-order curve estimation was performed for each nerve. Thus, compared to the first-order curve, the second-order curve was derived with a larger *R*^2^ value in all nerves, which showed that the second-order curve had a higher correlation than the first-order curve. Usually, the reference value of the CMAP amplitude greatly varies for each muscle, but it is generally 2–15 mV, with a maximum value.^[8]^ Since the axon number and muscle fiber density are limited, the second-order curve seems more reasonable.

The cutoff value was identified by drawing the ROC curve to predict the objective strength and CMAP amplitude by MMT. To distinguish between poor and fair grades, the femoral, peroneal, and tibial nerves are expected to have a CMAP of approximately 2.05, 1.7, and 2.3 mV, respectively, and a cutoff value of 17.3, 7.65, and 17.7 N-m or higher, respectively.

From Table 6, it is possible to predict the approximate CMAP amplitude and isometric strength during MMT. It is also possible to predict the MMT grade or isometric strength by considering only the CMAP amplitude. Beasley^[24]^ compared the isometric strength of the KE of normal subjects and polio patients, reporting that the good and fair grades were approximately 75% and 50%, respectively, of the normal grade. Furthermore, Kendall et al^[25]^ subdivided the MMT grades based on this and reported the theoretical percentage for easy prediction from zero to normal (zero, 0%; trace, 5%; poor−, 10%; poor, 20%; poor+, 30%; fair−, 40%; fair, 50%; fair+, 60%; good−, 70%; good, 80%; and good+, 90%). Bohannon compared the actual percentage of KE isometric strength to normal for each manual strength evaluation in patients with spinal cord injury, fracture, Guillain–Barré syndrome, peripheral neuropathy, root neuropathy, and muscular dystrophy, reporting that the good, fair, poor, and trace grades were 53.8%, 30.4%, 8.42%, and 1.83%, respectively, of the normal grade.^[26]^ In the current study, the good and fair grades were 48.9% and 31.0%, respectively, of the normal grade for the KE, in line with the Bohannon study. For the ADF, however, the good was 68% and the fair was 33.9% compared to normal, and for the ankle plantarflexor, the good was 54% and the fair was 31.3% compared to normal. Only the good grade in the ADF was more consistent with the study by Beasley.

For severe peripheral nerve damage due to multiple injuries, such as CES damage, MMT should be performed accurately. Nevertheless, a more objective examination is required when maximum patient cooperation is infeasible due to secondary gain, old age, or comorbidities. Thus, imaging tests such as MRI, sonography, and EMG are used accordingly. While imaging tests can detect changes in the nerves, they often fail to indicate the degree of nerve damage accurately. NCS can accurately determine the degree of nerve damage, but for the results to be clinically meaningful there must be an accurate correlation with actual lower extremity muscle weakness, as well as a criteria for making such judgments. Thus, this study aimed to suggest such relevant guidelines. Based on the data of this study, it is possible to identify the exact condition, objectively evaluate the degree of paralysis and disability, and determine quantitative muscle strength from MMT to establish an appropriate rehabilitation treatment plan.

## 5. Limitations

There are several limitations to this study. First, the number of target patients was small, making it impossible to analyze the correlations by sex and age. There is a notable difference between men and women, and in the elderly, sarcopenia and loss of muscle mass occur. McCormick and Vasilaki^[27]^ reported that the regenerative ability was reduced, and the infiltration of adipose tissue and fibrous tissue increased in muscle tissue, resulting in increased muscle stiffness in the elderly. Larsson et al^[28]^ reported a decrease in the proportion of type II muscle fibers in the elderly. In other words, the density of muscle fibers changes according to age, which could affect the CMAP amplitude. In future studies, it will be necessary to compare the associations by sex or age, and a large amount of data will be needed. Additionally, for an accurate comparison of muscle mass, only BMI and body weight were used to correct gender and age differences in muscle mass. It will be necessary to conduct research that reflects individual whole-body muscle mass measured by dual-energy X-ray absorptiometry.

Second, some of the enrolled patients underwent electrodiagnostic tests after a short hospitalization following long-term surgery due to serious trauma or degenerative disease. In the elderly, generalized weakness may have existed a short period after surgery, or the IST measured may have been less than the maximum strength due to pain at the surgical site. In a further study, adequate pain control will be needed in patients with postoperative pain, and ISTs should be conducted after improving generalized weakness.

## 6. Conclusion

In this study, the isometric strength of the KE, ADF, and ankle plantarflexor and the CMAP amplitude of the electrophysiologic parameters VM, TA, and GCM in patients with CES were correlated, and a significant correlation with MMT grade was also identified. Strength and MMT grade may be underestimated depending on the patient’s level of cooperation; thus, electrophysiological indicators are considered useful for objective evaluation. The method presented in this study is expected to help objective evaluation of patients who seek secondary gains as compensation issues are involved or patients with mental disorders, old age, or underlying diseases. Furthermore, it may contribute to establishing an objective rehabilitation treatment plan according to the degree of damage.

## Author contributions

**Conceptualization:** Jun-Hyeong Han, Ji-Young Lee, Dong Hyuk Yun, Chang-Won Moon, Kang Hee Cho.

**Data curation:** Jun-Hyeong Han, Chang-Won Moon.

**Formal analysis:** Jun-Hyeong Han, Ji-Young Lee, Dong Hyuk Yun, Chang-Won Moon, Kang Hee Cho.

**Investigation:** Jun-Hyeong Han, Chang-Won Moon, Kang Hee Cho.

**Methodology:** Jun-Hyeong Han, Chang-Won Moon, Kang Hee Cho.

**Project administration:** Kang Hee Cho.

**Supervision:** Chang-Won Moon, Kang Hee Cho.

**Validation:** Jun-Hyeong Han, Kang Hee Cho.

**Visualization:** Jun-Hyeong Han,

**Writing – original draft:** Jun-Hyeong Han, Chang-Won Moon, Kang Hee Cho, Chang-Won Moon, Kang Hee Cho.

**Writing – review & editing:** Jun-Hyeong Han, Chang-Won Moon, Kang Hee Cho, Chang-Won Moon, Kang Hee Cho.

## Correction

The funding information for this article was updated from “The authors have no funding and conflicts of interest to disclose” to “This work was supported by research fund of Chungnam National University.”
